# Inter- and intraspecific genetic and morphological variation in a sibling pair of carabid species

**DOI:** 10.1186/1746-1448-3-4

**Published:** 2007-04-24

**Authors:** Hilde Dhuyvetter, Jean-Pierre Maelfait, Konjev Desender

**Affiliations:** 1 Entomology Department, Royal Belgian Institute of Natural Sciences, Vautierstreet 29, Brussels, Belgium; 2 Instituut voor natuur- en bosonderzoek, Brussels, Belgium

## Abstract

**Background:**

*Pogonus littoralis *and *Pogonus chalceus *are very close related species with quite different ecological preferences within salt marshes. We study the evolutionary processes in and between these presumably young species. Therefore, we compare the variation in ecologically relevant characters and the genetic variation within one of the species (intraspecific differentiation) with the variation of the two types of characters between the two species (interspecific variation). Data are compared between two independent sets of populations, one set at a small geographical scale (the ecologically diverse Guérande area in France) and the other set at a Atlantic-Mediterranean scale.

**Results:**

Body and relative wing size and *IDH1 *allozyme data show that the intraspecific variation in *P. chalceus *is high and in the same range as the interspecific variation (*P. chalceus *versus *P. littoralis*). Based on neutral markers (other allozymes and mitochondrial DNA) on the other hand, the intraspecific variation in *P. chalceus *is much lower in comparison to the interspecific variation.

**Conclusion:**

The different ecotypes in the highly polytypic species *P. chalceus *are as highly differentiated in ecological characters as true species, but are not recognised as such by screening neutral DNA polymorphisms. This can be interpreted as a case of ongoing speciation driven by natural selection adapting each ecotype to its respective ecological niche. The same ecological process can be recognised in the differentiation between the two sister species, where en plus reproductive isolation between the two gene pools occurred, allowing independent drift and mutation accumulation in neutral genetic characters.

## Background

*Pogonus chalceus *is a wing polymorphic beetle with extremely variable wing size from short to completely developed wings, with all possible intermediates [1]. A recent study presented population genetic results on *P. chalceus *(Marsham, 1802) beetles from the Guérande salt-fields on the French Atlantic coast, based on allozymes and microsatellites, as well as results on wing and body size [2]. In the unique man made Guérande salt-fields, two contrasting habitat types are found mixed on a microscale in hundreds of replicates (sea canal versus salt extraction ponds). Body, relative wing size and *IDH1 *allozyme alleles are strongly divergent between these two contrasting microhabitats; divergent selection led to two clearly distinguishable ecotypes, respectively adapted to canal and pond habitat. Comparisons between the Guérande region (microscale) and populations along the Atlantic coast (macroscale) confirmed the generality of the hypothesis regarding ecological processes responsible for this differentiation: habitat stability [2]. The Guérande ecotypes are also slightly differentiated based on neutral molecular markers (microsatellites and allozymes), suggesting that partial barriers to gene flow between the two ecotypes are present. Previous work on a wide range of taxa has demonstrated that strong natural selection can lead to divergence in spite of gene flow [3-7]. Our Guérande results can therefore be interpreted as a case of ongoing speciation driven by natural selection adapting each ecotype to its respective ecological niche, i.e. species in *status nascenti *(see also [8,9]).

In the same Guérande region and along the European Atlantic and Mediterranean coast, another *Pogonus *species, *P. littoralis *(Duftschmid, 1812) lives in a third kind of microhabitat: unvegetated, temporary dry salt marsh ponds or creeks, where it lives between cracks in humid sea clay. This species is, in contrast to *P. chalceus*, constantly macropterous, always with maximally developed wings and functional flight musculature [10]. The beetle is highly mobile because it regularly has to move between temporarily dry salt marsh ponds and creeks during its life cycle. Both species can be hardly distinguished by external morphology (for example large individuals of *P*. *chalceus *versus small *P. littoralis*) but have clearly distinguishable genitalia.

The data in this article are to some extent compiled from previous works [2,11,12]. Nevertheless, the novelty of this study lies in the fact for the first time the two carabid sister species are analyzed jointly allowing for valuable comparisons to be made. In this study, we will first compare the two ecotypes of *Pogonus chalceus *with the closely related species, *Pogonus littoralis *at a microscale (Guérande region). Therefore, we will use population data on wing and body size, *IDH1 *allozyme polymorphism as well as apparently neutral markers (other allozymes and mtDNA). We will also test if the microscale results are valid at a larger scale across Europe by means of an independent data set of different populations of both *Pogonus *species. In all of these cases, we will evaluate the contribution of intra population, inter population, inter ecotype and interspecific variation to the total variance.

## Results

### Body size

**Figure 1:**
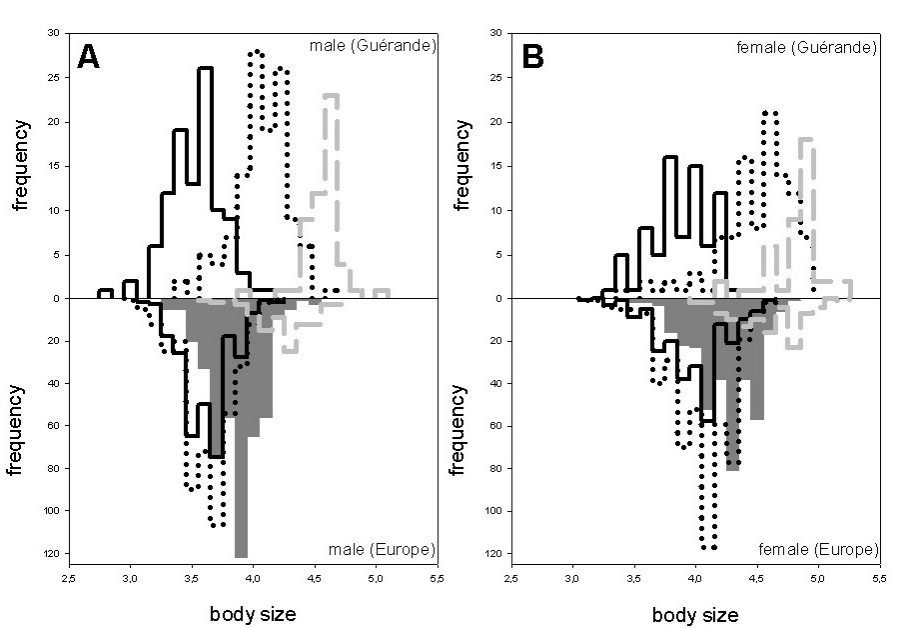
**Male (part A) and female (part B) body size frequency distributions**. In the upper part of A and B: Guérande canal populations (black solid line; *Pogonus chalceus*), pond populations (black dotted line; *Pogonus chalceus*) and *Pogonus littoralis *populations (gray dashed line). In the lower part of A and B: European stable populations (*P. chalceus*; black solid line), intermediate populations (*P. chalceus*; black dotted line), temporary Atlantic and Mediterranean populations (gray filled; *P. chalceus*) and *Pogonus littoralis *populations (gray dashed line). Figure legend text.

Fig. 1 shows male body size for both *P. chalceus *ecotypes and for the *P. littoralis *populations in the Guérande region [see also additional file 1]. Mean body size for the pond populations is small (3.56; range: 2.9–4.2), intermediate for the canal populations (4.08; 3.4–4.6) and high for the *P. littoralis *populations (4.69; range: 3.9–5.1). Body size values for the females show a similar pattern but are always larger than male body sizes. Mean female body size is 3.91 for the canal populations (range: 3.4–4.3), 4.52 for the pond populations (range: 3.4–5.0) and 4.89 for the *P. littoralis *populations (range: 4.3–5.3).

Fig. 1 also shows male and female body sizes for the *P. chalceus *ecological groups and for the *P. littoralis *populations on a European scale [see also additional file 2]. Mean male body size is small for the stable (3.68; range: 3.2–4.3) and intermediate *P. chalceus *populations (3.68; range 3–4.3), somewhat higher for the temporary populations (mean: 3.92; range: 3.4–4.6) and high for the *P. littoralis *(mean: 4.32; range: 3.6–4.8) ones. Body size values of females show again a similar pattern and are always larger than male body sizes. Mean female body size is 4.04 for the stable *P. chalceus *populations (range: 3.2 to 4.6) compared to 4.11 for the intermediate populations (range: 3.1 to 4.7) and 4.3 for the temporary *P. chalceus *populations (range: 3.4 to 4.8) and 4.6 for the *P. littoralis *populations (4.1–5.2).

In the Guérande region and considering the two species (nested design ANOVA; six *P. chalceus *populations (canals and ponds pooled) versus three *P. littoralis *populations, the major part of variance (based on body size) is found among species (Table 1; 74.24% for males and 51.96% for females). If we consider three groups (three canal populations (*P. chalceus*), three pond populations (*P. chalceus*) and three *P. littoralis *populations, the major part of variance is even more pronouncedly found among groups (84.96% for males and 72.08% for females). Variance among populations within groups considering three groups instead of two drops from 17.5 to 2.35% for males and from 29.57 to 4.28% for females. This indicates that this variance was almost completely due to the differences in body size between populations of *P. chalceus *from different microhabitats. All variance components are statistically significant.

**Table 1:** Analysis of variance (nested design ANOVA) based on male or female body size in two regions: Guérande microscale and Europe macroscale

region	groups	source of variation	% var male	% var female
Guérande	*P. chalceus*/*P. littoralis*	among groups	74.24	51.96
		among populations within groups	17.50	29.57
		within populations	8.26	18.47
	ponds/canals/*P. littoralis*	among groups	84.96	72.08
		among populations within groups	2.35	4.28
		within populations	12.69	23.63
				
Europe	*P. chalceus*/*P. littoralis*	among groups	68.37	48.37
		among populations within groups	10.13	12.29
		within populations	21.51	39.35
	stable/intermediate/temporary/*P. littoralis*	among groups	49.39	33.87
		among populations within groups	5.00	7.61
		within populations	45.62	58.53

On a European scale and considering the two species (25 *P. chalceus *populations versus six *P. littoralis*), the major part of variance (based on body size) is found among species (Table 1; 68.37% for males and 48.37% for females). If we consider four ecological groups (14 temporary (*P. chalceus*), five intermediate (*P. chalceus*), six stable (*P. chalceus*) and five *P. littoralis*), the variance among groups drops (49.39% for males and 33.87% for females) and the variance within populations augments (45.62% for males and 58.53% for females). Variance among populations within groups considering four groups instead of two drops a little from 10.13 to 5% for males and from 12.29 to 7.61% for females. All variance components are statistically significant.

### Relative wing size

**Figure 2:**
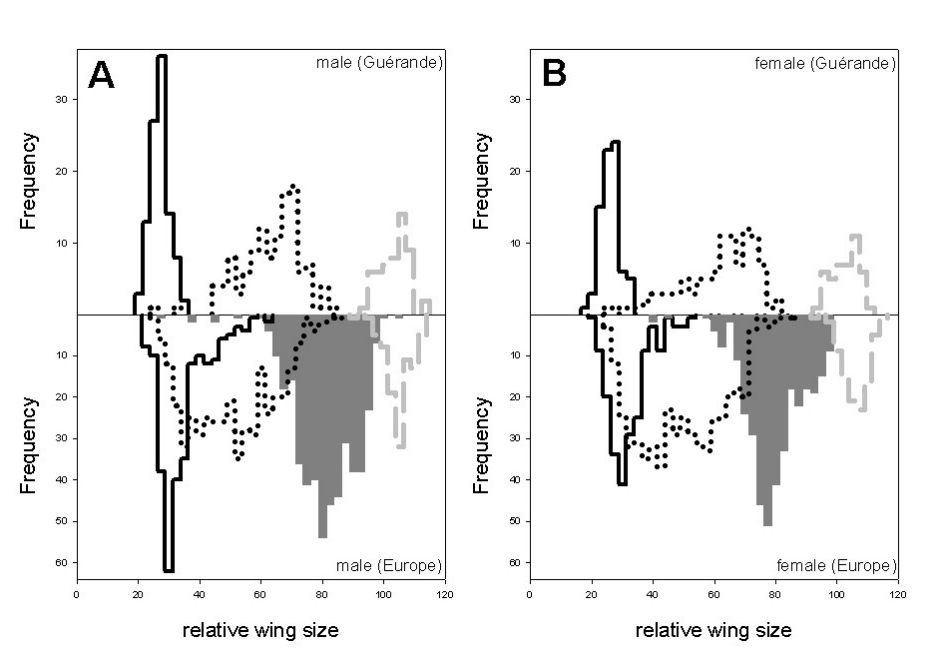
**Male (part A) and female (part B) relative wing size frequency distributions**. In the upper part of A and B: Guérande canal populations (black solid line; *Pogonus chalceus*), pond populations (black dotted line; *Pogonus chalceus*) and *Pogonus littoralis *populations (gray dashed line). In the lower part of A and B: European stable populations (*P. chalceus*; black solid line), intermediate populations (*P. chalceus*; black dotted line), temporary Atlantic and Mediterranean populations (gray filled; *P. chalceus*) and *Pogonus littoralis *populations (gray dashed line).

Fig. 2 shows male and female relative wing sizes for both *P. chalceus *microhabitats and for the *P. littoralis *populations in the Guérande region [see also additional file 1]. Mean male relative wing size for the canal populations is small (28.19; range: 20–35%), intermediate for the ponds (mean: 64.24; range: 25–82.5%) and high for the *P. littoralis *populations (mean: 103.59; range: 92.5–112.5%). Relative wing size values of females show a similar pattern and are not larger than male relative wing sizes. Mean female relative wing size for the canals is 26.93 (range: 17.5–32.5%), 62.31 for the ponds (range: 25–80%) and 103.04 for the *P. littoralis *populations (range: 92.5–110%).

Fig. 2 also shows male and female relative wing size in ecological groups of *P. chalceus *and of *P. littoralis *populations on a European scale [see also additional file 2]. Mean male relative wing size is small for the populations of the old, highly stable salt marsh areas (35.28; range: 22.5–62.5%), some higher for the populations of the salt marshes of intermediate stability (mean: 51.07; range: 25–85%), higher for the populations of the small, unstable areas (mean: 82.23, range: 27.5–105%) and very high for the *P. littoralis *populations (mean: 106.16; range: 90–112.5%). Relative wing size values of females show a similar pattern and are not larger or smaller than male relative wing sizes. Mean female relative wing size for the stable populations is 33.43 (range: 20–82.5%) compared to 49.91 for the populations of intermediate stability situations (range: 25–85%), 80.41 for the temporary populations of the highly unstable salt marshes (range: 40–97.5%) and 107 for the *P. littoralis *populations (range: 92.5–115%).

In the Guérande region and considering the two species (nested design ANOVA; six *P. chalceus *populations (canals and ponds pooled) and three *P. littoralis *populations), the major part of variance (based on relative wing size) is found among species (Table 2; 78.84% for males and 80.37% for females). If we consider three groups (three canal populations (*P. chalceus*), three pond populations (*P. chalceus*) and three *P. littoralis *populations), the major part of variance is even more clearly found among groups (95.48% for males and 93.92% for females). Variance among populations within groups considering three groups instead of two drops from 18.64 to 0.64% for males and from 16.21 to 0.8% for females. This indicates that this variance is almost completely due to the differences in relative wing size between populations of *P. chalceus *from different microhabitats. All variance components are statistically significant.

**Table 2:** Analysis of variance (nested design ANOVA) based on male or female relative wing size in two regions: Guérande microscale and Europe macroscale

	groups	source of variation	% var male	% var female
Guérande	*P. chalceus*/*P. littoralis*	among groups	78.84	80.37
		among populations within groups	18.64	16.21
		within populations	2.52	3.43
	ponds/canals/*P. littoralis*	among groups	95.48	93.92
		among populations within groups	0.64	0.80
		within populations	3.88	5.58
				
Europe	*P. chalceus*/*P. littoralis*	among groups	57.61	60.16
		among populations within groups	36.11	32.80
		within populations	6.28	7.05
	stable/intermediate/temporary/*P. littoralis*	among groups	82.22	82.58
		among populations within groups	6.34	6.89
		within populations	11.45	10.54

On a European scale and considering the two species (25 *P. chalceus *populations versus six *P. littoralis *populations), the major part of variance (based on relative wing size) is found among species (Table 2; 57.61% for males and 60.16% for females). If we consider four ecological groups (14 temporary (*P. chalceus*), five intermediate (*P. chalceus*), six stable (*P. chalceus*) and six *P. littoralis *populations), the major part of variance is again even more pronounced among groups (82.22% for males and 82.58% for females). Variance among populations within groups considering four groups instead of two drops from 36.11 to 6.34% for males and from 32.8 to 6.89% for females. This indicates that this variance is again almost completely due to the differences in relative wing size between populations of *P. chalceus *from different ecological or salt marsh area stability groups. All variance components are statistically significant.

### IDH1 allozyme marker

**Figure 3:**
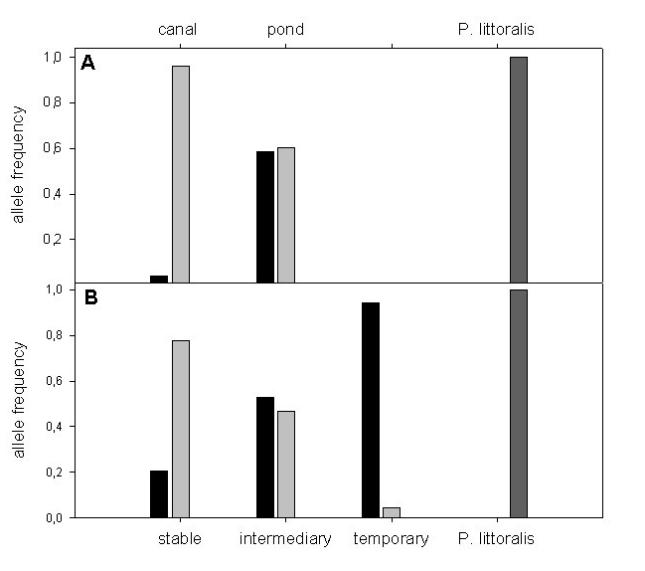
**Allele frequencies for *IDH1 *in the Guérande region (part A; cnals (P. chalceus) and ponds (*P. chalceus*) and *P. littoralis*)**. Allele frequencies for *IDH1 *on a European scale (part B; stable (*P*. *chalceus*) and intermediate (*P. chalceus*), temporary and *P. littoralis*). *Idh1-2*: black, *Idh1-4*: light gray; *Idh1-6*: dark gray.

**Figure 4:**
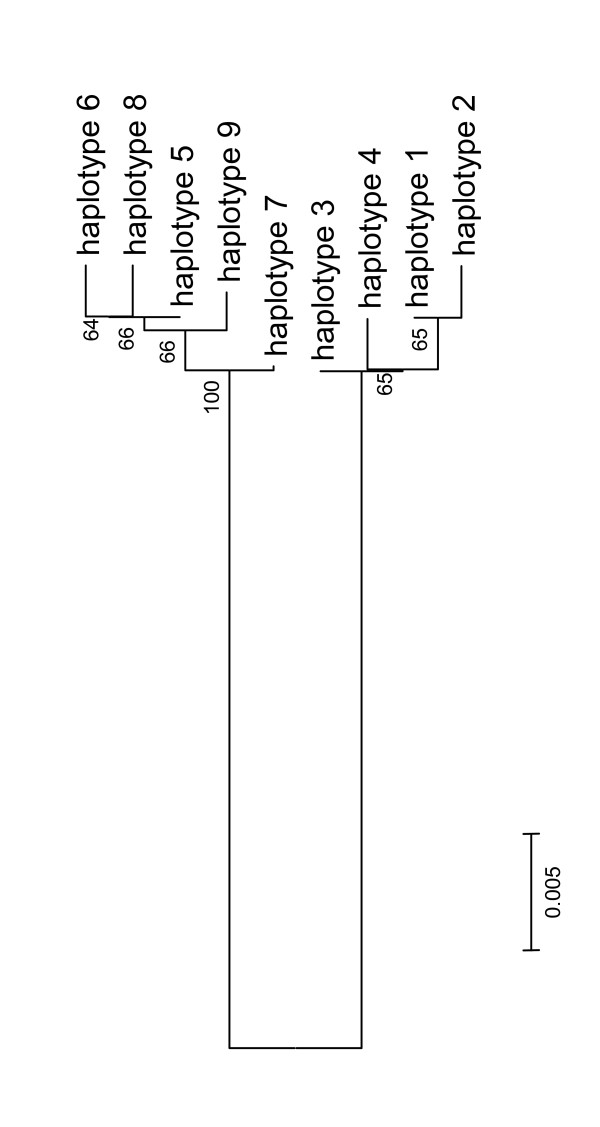
**Neighbour joining tree based on COI haplotypes described in Table 2**. Haplotype 1–4 are found in *P. chalceus *and haplotype 5–9 in *P. littoralis*. Bootstrap values are indicated (1000 replicates).

In Guérande, both *Idh1-2 *and *Idh1-4 *alleles are frequent in ponds, whereas canals are nearly fixed at *Idh1-4 *(Fig. 3A) [see also additional file 1]. *P. littoralis *populations are fixed at the *Idh1-6 *allele. Allele *Idh1-1*, *Idh1-3*, and *Idh1-5 *are very rare in *P. chalceus *and therefore not shown in Figure 4. Considering the two species (AMOVA; six *P. chalceus *populations (canals and ponds pooled) and three *P. littoralis*), the major part of variance (based on *IDH1*) is found among groups (Table 3; 61.93%). If we consider three groups (three canal populations (*P. chalceus*), three pond populations (*P. chalceus*) and three *P. littoralis *populations, the major part of variance is still found among groups (64.25%). Variance among populations within groups considering three groups instead of two drops from 11.49 to 0.1%. This indicates that this variance is almost completely due to differences in *IDH1 *between populations of *P. chalceus *from different microhabitats. All variance components are statistically significant.

**Table 3:** Analysis of molecular variance (AMOVA) based on *IDH1 *allozyme or 4 neutral allozymes in two regions: Guérande microscale and Europe macroscale

	groups	source of variation	% var *IDH1*	% var allo
Guérande	*P. chalceus*/*P. littoralis*	among groups	61.93	58.43
		among populations within groups	11.49	3.39
		within populations	26.57	39.18
	ponds/canals/*P. littoralis*	among groups	64.25	41.99
		among populations within groups	0.1	4.98
		within populations	35.65	53.02
				
Europe	*P. chalceus*/*P. littoralis*	among groups	62.29	36.49
		among populations within groups	12.26	13.70
		within populations	25.44	49.81
	stable/intermediate/temporary/*P. littoralis*	among groups	53.26	19.05
		among populations within groups	2.07	15.71
		within populations	44.67	65.24

At a European scale, both *Idh1-2 *and *Idh1-4 *alleles are frequent in the intermediate stability populations, whereas the temporary populations are nearly fixed at the *Idh1-2 *allele and the stable populations at the *Idh1-4 *allele (Fig. 3B) [see also additional file 2]. *P. littoralis *populations are fixed at the *Idh1-6 *allele. Considering two groups (25 *P. chalceus *populations versus six *P. littoralis *populations, the major part of variance (based on *IDH1*) is found among groups (Table 3; 62.29%). If we consider four ecological groups (14 temporary (*P. chalceus*), five intermediately stable (*P. chalceus*), six highly stable (*P. chalceus*) and six *P. littoralis *populations, the major part of variance is somewhat lower but is still found among groups (53.26%). Variance among populations within groups, considering four groups instead of two, drops from 12.26 to 2.07%. This indicates that this variance is almost completely due to differences in *IDH1 *between populations of *P. chalceus *from different ecological groups, as *P. littoralis *is fixed in a different allele. All variance components are statistically significant.

### Other allozymes

The number of studied individuals and allozyme allele frequencies for each population in the Guérande region is given in additional file 3. In the Guérande and considering two groups (AMOVA; six *P. chalceus *populations (canals and ponds) versus three *P. littoralis *populations), the major part of variance (based on four neutral allozymes) is found between species (Table 3; 58.43%). If we consider three groups (three canal populations (*P. chalceus*), three pond populations (*P. chalceus*) and three *P. littoralis *populations, the variance among groups drops to 41.99% and the major part of variance is now found within populations (53.02%). Variance among populations within groups considering three groups instead of two remains in the same range (3.39% for two groups compared to 4.98% for three groups).

The number of studied individuals and allozyme allele frequencies for each population at a European scale is given in additional file 4. At a European scale and considering two groups (AMOVA; 25 *P. chalceus *populations and six *P. littoralis *populations), the major part of variance (based on four neutral allozymes) is found within populations (Table 3; 49.81%) and among groups (36.49%). If we consider four ecological groups (14 temporary (*P. chalceus*), five intermediately stable (*P. chalceus*), six highly stable (*P. chalceus*) and six *P. littoralis *populations, the variance among groups drops to 19.05% and the major part of variance is still found within populations (65.24%). Variance among populations within groups considering four groups instead of two remains in the same range (13.70% for two groups compared to 15.71% for four groups). All variance components are statistically significant.

### Mitochondrial DNA

The 459-bp COI mitochondrial sequences revealed two haplotypes in the Guérande region. Haplotype one was shared by individuals of both canal and pond ecotype. Haplotype two was exclusive to the canal ecotype (Table 4). The 497-bp 16S sequences revealed only one haplotype in the Guérande region (Table 5).

**Table 4:** 459 bp of COI sequenced for 90 individuals of *P. chalceus *and 22 of *P. littoralis *

GATTAGTTCCTTTAATATT**x**AGCACC**x**GATATAGC**x**TTTCCTCGAATAAATAATATAAGTTTTTGA**x**TATTACCTCCTTC**x**TTAACACTACTTTTAATAAG**x**AG**x** . . . . . . . . . . . . . . . . .1. . . . . . .2. . . . . . . .3 . . . . . . . . . . . . . . . . . . . . . . . . . . 4 . . . . . . . . . . . 5. . . . . . . . . . . . . . . . . . 6. . 7 ATGGTAGAAAGAGG**x**GCTGGTACAGGATGAACTGT**x**TA**x**CCTCC**xx**TATC**x**TCTG**x**TATTGCACATAGAG**x**GGCTTCAGTAGATTTAGC**x**ATTTTTAGTCTTCATT . . . . . . . . . . . . . . . 8. . . . . . . . . . . . . . . . . . 9 . .10 . . . 1112 . . 13 . . 14 . . . . . . . . . . . .15 . . . . . . . . . . . . . . . 16 . . . . . . . . . . . . . TAGCAGG**x**GT**x**TCTTCAATTTT**x**GGAGCTGT**x**AATTTTATTACAACTATTATTAATATACGATCA**x**TTGGAATAACATTTGA**c**CGAATACCTTTATTTGT**x**TGATC . . . . . . 17 . 18 . . . . . . . . 19 . . . . . . 20 . . . . . . . . . . . . . . . . . . . . . . . . . . . . . 21 . . . . . . . . . . . . . . 22 . . . . . . . . . . . . . . .23 . . . TGTAGGAATTACTGCTTTACTTTTATTATTATCATTACCAGTTTTAGCTGGAGCAATTAC**x**ATACTTTTAAC**x**GATCGAAATTTAAATAC**x**TC**x**TTTTTTGACCC**x** . . . . . . . . . . . . . . . . . . . . . . . . . . . . . . . . . . . . . . . . . . . . . . . . . . . . . .24 . . . . . . . . .25 . . . . . . . . . . . . . . 26 . 27. . . . . . . . 28 . GC**x**GGAGGAGGAGA**x**CC**x**ATTTTATA**x**CAACA . .29 . . . . . . . . . 30 . .31 . . . . . . 32 . . . .																																
	Haplotype sequence information																															

Haplotype No.	1	2	3	4	5	6	7	8	9	10	11	12	13	14	15	16	17	18	19	20	21	22	23	24	25	26	27	28	29	30	31	32

1	A	T	A	C	T	C	A	A	A	T	T	T	T	C	G	T	A	T	A	A	A	C	T	A	T	A	A	T	T	C	A	C
2	A	T	A	C	T	C	A	A	A	T	T	T	T	C	G	T	A	C	A	A	A	C	T	A	T	A	A	T	T	C	A	C
3	A	T	A	C	T	C	A	A	A	T	T	T	T	C	G	T	A	T	A	A	A	T	T	A	T	A	A	T	T	C	A	C
4	A	T	C	C	T	C	A	A	A	T	T	T	T	C	G	T	A	T	A	A	A	T	T	A	T	A	A	T	T	C	A	C
5	A	A	A	T	A	T	T	T	T	C	C	C	A	T	T	A	G	T	G	T	G	T	C	T	T	T	T	A	A	T	T	T
6	A	A	A	T	A	T	T	T	T	C	C	C	A	T	T	A	G	T	G	T	G	T	C	T	C	T	T	A	A	T	T	T
7	A	A	A	T	A	T	T	T	T	C	C	C	A	T	T	A	A	T	G	T	G	T	C	T	T	T	T	A	A	T	T	T
8	G	A	A	T	A	T	T	T	T	C	C	C	A	T	T	A	G	T	G	T	G	T	C	T	T	T	T	A	A	T	T	T
9	A	A	A	T	A	T	T	T	T	C	C	C	A	T	T	A	G	T	G	T	T	T	C	T	T	T	T	A	A	T	T	T

	FREQUENCY OF HAPLOTYPES																															
	
	*POGONUS CHALCEUS*														*POGONUS LITTORALIS*																	
																	
Haplotype No.	CANAL1	POND1	MOK	ZWC	HEI	OOS	NIE	CAN	SOM	MSM	GAC	GIR	CAMA	ALB	GUE1	ZWC	ROU															

1	8	6	6	7	7	6					2	7																				
2	3																															
3			1				7	5	6	4	1		6	6																		
4									1																							
5															5	3	6															
6																	1															
7																4																
8															1																	
9															1																	

Bold lower case letters show variable positions numbered from 1 to 32 which are not position numbers in the gene. Dots indicate invariable positions. The table shows the variable sites of the haplotypes. The table below shows the haplotype frequency in each population.

**Table 5:** 497 bp of 16S sequenced for 62 individuals of *P. chalceus *and 15 of *P. littoralis*.

TTTATCAAAAACATGTCTTTTTGAGTTTAATATAAAGTCTA**x**CCTGCCCACTGAAA**x**TTTTAAATGGCCGCAGTAATTTGACTGTGCAAAGGTAGCATAATCT . . . . . . . . . . . . . . . . . . . . . . . . . . . . . . . . . . . . .1 . . . . . . . . . . . . .2 . . . . . . . . . . . . . . . . . . . . . . . . . . . . . . . . . . . . . . . . . . TAGTTTTTTAATTGAAAGCTTGTATGAAAGGTTGGACGAGGTAAAATCTGTCTCTATTTAATTTA**x**ATTAGAATTTAATTTTTAAGTTAAAAAGCTTAAATTTT . . . . . . . . . . . . . . . . . . . . . . . . . . . . . . . . . . . . . . . . . . . . . . . . . . . . . . . . . . 3 . . . . . . . . . . . . . . . . . . . . . . . . . . . . . . . . . . TTTAAAAGACGAGAAGACCCTATAGAGCTTTATAATTTATTTAATATAATTAATTTAGATTTATTTATATTTTATT**x**TT**x**AAATTATTTTATTGGGGTAATAGA . . . . . . . . . . . . . . . . . . . . . . . . . . . . . . . . . . . . . . . . . . . . . . . . . . . . . . . . . . . . . . . . . . . . . .4 . 5 . . . . . . . . . . . . . . . . . . . . . . . AGATTAAAAAAATTCTTTTTTTTTATTTATATT**xx**TTTAT**x**TTTT**x**AATGATCCA**x**TTTTATTGATTATAAGATTAAGTTACCTTAGGGATAACAGCGTAATTTTT . . . . . . . . . . . . . . . . . . . . . . . . . . . . . .67 . . . .8 9 . . . 10 . . . . . . . .11. . . . . . . . . . . . . . . . . . . . . . . . . . . . . . . . . . . . . . . . . . . . . . TGGAGAGTTCAT ATCGATAAAAAAGTTTGCGACCTCGATGTTGGATTAAAGATTAGTTTAGGTGTAGAAGTTTAAA . . . . . . . . . . . . . . . . . . . . . . . . . . . . . . . . . . . . . . . . . . . . . . . . . . . . . . . . . . . . . . . . . . . . . . .														
	Haplotype sequence information													

Haplotype No.	1	2	3	4	5	6	7	8	9	10	11			

1	G	T	G	T	A	T	A	A		T	T			
2	G	T	G	T	A	T	A			T	T			
3	A	A	A	A	T	A	G	A	G	A	G			

	Frequency of haplotypes													
	
	*POGONUS CHALCEUS*											*POGONUS LITTORALIS*		
	
Haplotype No.	CANAL1	POND1	MOK	ZWC	HEI	OOS	NIE	SOM	MSM	CAMA	ALB	GUE1	ZWC	ROU

1	6	6	5	6	7	4	5	5	7	5	5			
2				1										
3												6	5	4

Bold lower case letters indicate variable positions numbered from 1 to 11 which are not position numbers in the gene. Dots indicate invariable positions. The table shows the variable sites 1–11 of the haplotypes. The table below shows the haplotype frequency in each population.

The 459-bp COI sequences included 32 variable sites on a European scale (29 parsimony informative) and revealed nine unique haplotypes (four for *P. chalceus *and five for *P. littoralis*, with no haplotype shared by the two species). Most haplotypes were exclusive to a particular sampling site, with the exception of haplotype one and three which appeared in eight different localities, and haplotype five, which was found in three localities (Table 4). Selective neutrality was confirmed for this gene (*P *> 0.1 for all test statistics with D* and F*).

**Figure 5:**
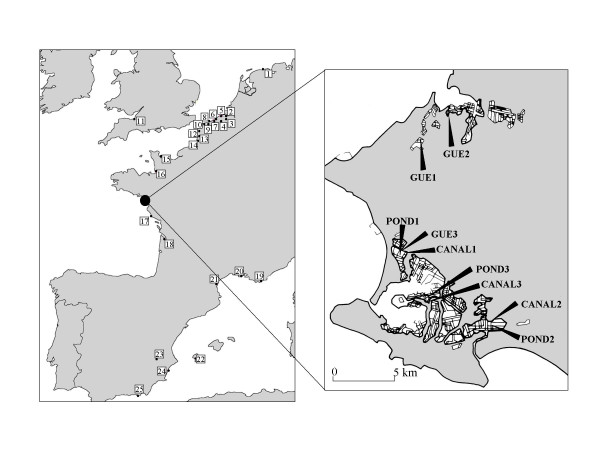
**Geographical distribution of the studied populations of *Pogonus chalceus *and *Pogonus littoralis***. The following population codes were used: 1:FRI; 2:BRA; 3:WAT; 4:MOK; 5:ZWC; 6:HEI; 7:LIS; 8:OOS; 9:NIE; 10:MOE; 11:SEA; 12:CAN; 13:AUT 14:SOM; 15: MSM; 16:VEY; 17:GAC; 18:GIR; 19:TOU; 20: CAM; 21:ROU; 22:IBI; 23:ALB; 24:MUR: 25:ALM. *P. chalceus *populations were sampled from all indicated numbers (1–25). *P. littoralis *were sampled in the populations with number 5, 13, 15, 19, 20 and 21. The detailed map is the Guérande region containing three studied *P. chalceus *pond populations (POND1, POND2, POND3), three *P. chalceus *canal populations (CANAL1, CANAL2, CANAL3) and three *P. littoralis *populations (GUE1, GUE2, GUE3).

The neighbour joining tree in Figure 5 shows that both species form clearly separated entities (high bootstrap values). Differences are found in 28 positions between haplotypes 5,6,8,9 (*P*. *littoralis*) and haplotypes 1 and 2 (*P. chalceus*; Table 4). 27 base differences are found between haplotypes 5,6,8,9 and haplotypes 3 and 4 (*P. chalceus*). 27 base differences are found between haplotype 7 (*P*. *littoralis*) and haplotypes 1,2 (*P. chalceus*) and 26 base differences with haplotypes 3,4 *(P. chalceus*). Intrapopulation differences in both *P. chalceus *and *P. littoralis *are very small (only a limited number of individuals studied) and between each haplotype there are only one to at most two bases different.

The 497-bp 16S sequences included 11 variable sites (9 parsimony informative) and revealed three unique haplotypes (Table 5; two for *P. chalceus *and one for *P. littoralis*, with no haplotype shared by the two species). Only one haplotype was exclusive to a particular sampling site (haplotype two). Haplotype one appeared in all 11 *P. chalceus *localities, and haplotype three appeared in all three *P. littoralis *sites. Selective neutrality was confirmed for this gene (*P *> 0.1 for all test statistics with D* and F*). Both species form, as in the case for COI, clearly separated entities (Table 5). 10 base differences are found between haplotype 3 (*P*. *littoralis*) and haplotypes 1 and 2 (*P. chalceus*). Interpopulation differences in both *P. chalceus *are very small (between the two haplotypes there is only one base different). There were no interpopulation differences found in *P. littoralis*.

## Discussion

*Pogonus littoralis *and *Pogonus chalceus *are closely related species, sometimes relatively hard to identify without dissection of the genitalia. We are interested to study the evolutionary processes in and between these presumably young species. We therefore compare the degree of intraspecific variation (in ecological groups of *P. chalceus*) and the degree of interspecific variation (*P. chalceus *versus *P. littoralis*) between a variety of morphological characteristics and molecular markers. In all of these cases, we did this with an ANOVA splitting up the total variance among groups, among populations within groups and within populations (Table 6).

**Table 6:** Summary of analysis of variance for body size, wing size, *IDH1 *and allozymes

region	source of variation	% var body size male	% var wing size male	% var *IDH1*	% var allo
Guérande	among groups (*P. chalceus *vs *P. littoralis*)	74.24	78.84	61.93	58.43
	among populations within groups	17.50	18.64	11.49	3.39
	within populations	8.26	2.52	26.57	39.18
Europe	among groups (*P. chalceus *vs *P. littoralis*)	68.37	57.61	62.29	36.49
	among populations within groups	10.13	36.11	12.26	13.70
	within populations	21.51	6.28	25.44	49.81
					
Guérande	among groups (2 ecotypes vs *P. littoralis*)	84.96	95.48	64.25	41.99
	among populations within groups	2.35	0.64	0.1	4.98
	within populations	12.69	3.88	35.65	53.02
Europe	among groups (3 ecolog groups vs *P. littoralis*)	49.39	82.22	53.26	19.05
	among populations within groups	5	6.34	2.07	15.71
	within populations	45.62	11.45	44.67	65.24

At both geographical scales and considering two groups (*P. chalceus *populations versus *P. littoralis *populations), a very large part of the total variance (based on body size, relative wing size, *IDH1 *and four neutral allozymes) is found between species (summary in Table 6). The study of the two mitochondrial genes also shows that both species form clearly separated entities. It is clear that relative wing size differences as well as genetic differences between the sister species *P. chalceus *and *P. littoralis *(interspecific) in this study are very marked and allow an easy species recognition.

Body size, relative wing size and *IDH1 *allozyme data in the beetle *P. chalceus *are also strongly divergent between contrasting microhabitats (intraspecific: two ecotypes in Guérande) as well as between three ecological groups at macroscale (highly stable versus intermediately stable and temporary populations; [2] and this study). If we consider four groups on a macroscale (3 groups in *P. chalceus *+ 1 group of *P. littoralis*) or three groups on a microscale (2 ecotypes in *P. chalceus *+ 1 group of *P. littoralis*), the variance among populations within groups drops drastically as compared to the analysis of two groups (all *P. chalceus *populations versus *P. littoralis*; based again on body size, relative wing size and *IDH1*; summary in Table 6). This study clearly shows that the intraspecific variation based on those three characteristics in *P. chalceus *is very high and in the same order of magnitude as the degree of interspecific variation (*P. chalceus *versus *P. littoralis*). We have suggested earlier that this huge phenotypic and *IDH1 *divergence in *P. chalceus *has been driven by divergent natural selection [2]. As relative wing size is to a large extent genetically determined [1], this indeed suggests divergent selection between populations. And the observation that the *IDH1 *locus screened within our samples shows alelic differences between habitats strongly suggests a locus undergoing evolution through natural selection. Moreover, the canal and pond microhabitats differ from each other with respect to temperature, salinity and water level fluctuations [2]. Numerous studies based on allozymes have revealed patterns of allelic distribution associated with environmental factors, such as temperature and salinity [13,14]. Regarding the function of *IDH1*, the enzyme catalyses the rate-limiting step of the tricarboxylate cycle. Possible links with growth, however, are not direct and could be associated with the energy that is produced from the reaction. Divergent selection can lead to reproductive isolation and assortative mating and ultimately to speciation [8,15].

On the other hand, in a previously study was shown that *P. chalceus *ecotypes in the Guérande region were only slightly differentiated (based on allozyme and microsatellite markers) compared to the results based on adaptive characteristics [2]. The smaller degree of intraspecific divergence is also reflected in the mitochondrial data from this study. Moreover, allozyme and mtDNA data from this study show that the populations of *P. chalceus *are much more related to each other than to their sister species *P. littoralis *both on a micro- and macroscale. Often, little or no genetic divergence is found in neutral markers between ecologically and morphologically differentiated populations [3-5,7,16-18]. Our results can be interpreted as a case of ongoing speciation in *P. chalceus *where divergence reflects a balance between selection and gene flow (see also [2]). Several studies suggest that tital marshes may be an appropriate ecotone in which to search for instances of ecological speciation. The studied species show, as is the case in our study, distinct morphological differences despite little divergence in molecular markers [7,19-21].

In view of the above shown analogy between intra- and interspecific variation, it seems reasonable to assume that the same ecological adaptive bifurcation was also the first step in the speciation process of *P. chalceus *and *P. littoralis*. The speciation process was here fully accomplished by the reproductive isolation between the two groups, allowing independent drift and mutation accumulation in neutral genetic characters.

## Methods

### Sampling

*P. chalceus *populations from three different sites in the Guérande region are analysed (microscale; Fig. 5; see also [2]). Each site consists of two drastically differing microhabitats, situated only 10–20 metres from each other and separated by one or two dikes. We compare *P. chalceus *populations from three canals (CANAL1; CANAL2; CANAL3: Fig. 5) to three adjacent pond populations (POND1; POND2, POND3; see also [2]). Furthermore, we analyse Guérande *P. littoralis *populations from three different sites (GUE1, GUE2, GUE3; Fig. 5; see also [12]) nearby the aforementioned *P. chalceus *population couples.

The two related species are also studied on a macroscale with completely independent population samples (Guérande populations not included). Data on *P. chalceus *populations from the Netherlands (FRI), Belgium (BRA, WAT, MOK, ZWC, HEI, LIS, OOS, NIE, MOE), England (SEA), France (CAN, AUT, SOM, MSM, VEY, GAC, GIR, TOU, CAM, ROU), and Spain (IBI, ALB, MUR, ALM; Fig. 5; see also [11,22]. For *P. littoralis*, six populations are analysed here, five of them from France (AUT, MSM, TOU, CAM, ROU; [12]). From these sites in France, we also sampled *P. chalceus *populations (see above). In Belgium, *P. littoralis *is critically endangered and the previous record went back to 1956 and was from Ostend [23]. Recently, a supposed new *P. littoralis *population has been discovered in Belgium and is also included here (Fig. 5; ZWC). Populations of *P. chalceus *on a European scale were assigned to belong to one of three different salt marsh area stability types: temporary (BRA, WAT, MOK, HEI, LIS, OOS, MOE, TOU, CAM, ROU, IBI, ALB, MUR, ALM), intermediate (ZWC, NIE, MSM, FRI, AUT) and stable (SEA, CAN, SOM, VEY, GAC, GIR; see also [2,11]. Temporary populations of *P. chalceus *are situated in the Mediterranean part of Europe or occur in small (<4 ha) and young (<400 years) Atlantic salt marshes. Stable and intermediate populations live in larger marshes situated along the Atlantic coast. The only difference between both salt marsh areas is their estimated age (Stable: >1000 years; Intermediate: between 400–1000 years). The age of salt marshes was estimated using historical information [24-27].

### Morphological analysis

Body size (elytral length) and wing size were measured by means of a calibrated ocular under a binocular microscope. Generally, carabid wing size follows an allometric relationship with body size. [28] developed an index that corrects for this allometry, i.e. percentage of maximal realisable relative wing size. Relative wing size is wing length × width divided by elytral length × width. Relative wing size is then expressed as a percentage of the maximal wing size for a beetle of a given size. This index was shown to be an unbiased estimator for comparing different individuals, populations and species of carabid beetles [28]. In ground beetles, females are generally larger than males. Therefore, we analyse male and female body sizes separately. To be complete, we analyse female and male relative wing size also separately. Body size and relative wing size are compared between species and populations with ANOVA's. Total variance is partitioned among groups (species or species and ecotypes), among populations within groups, and within populations by carrying out a nested design ANOVA using STATISTICA (version 7.1; StatSoft Inc., Tulsa, UK) on both a micro- and macroscale.

### Genetic divergence

Data are used from five polymorphic enzymes: aldehyde oxidase (*AO*, E.C. 1.2.3.1), glucose-6-phosphate isomerase (*GPI*, E.C. 5.3.1.9), isocitrate dehydrogenase 1 and 2 (*IDH1*, *IDH2*, E.C. 1.1.1.42), phosphoglucomutase (*PGM*, E.C. 2.7.5.1.). Protocols of electrophoresis are provided by [29]. Earlier work showed that one locus (*IDH1*) was non-neutral and we will always analyse it separately [11].

Departures from Hardy-Weinberg equilibrium expectation were tested with an exact test using the GENEPOP software (Version 3.2; [30]). Significance levels were adjusted by using sequential Bonferroni correction. Similarly as in the analyses for body and wing size, total genetic variance is partitioned among groups (species, ecotypes), among populations within groups, and within populations by carrying out a hierarchical analysis of molecular variance (AMOVA) using ARLEQUIN (version 3.000; [31]) on both a micro- and macroscale.

PCRs for nucleotide sequencing of COI utilized primers C1-J-1718 and C1-N-2191 and for 16S we utilized primers LR-J-1307 and LR-N-13398 [32]. DNA amplification reactions were performed in a 25 μL final volume. Each reaction mix contained 5 μL of extract, 1× buffer (Sigma), 1.5 mM MgCl_2_, 200 μM of each dNTP, 0.4 μM of each primer and 0.6 U *RedTaq *DNA polymerase (Sigma). Initial denaturation was for 2 min at 95°C, followed by 35 cycles of 1 min at 95°C, 1 min 30 s at 48°C (and 46°C for 16S), and 2 min at 72°C; 9 min at 72°C completed the program. The reaction was purified with columns following manufacturer's recommendations. Sequencing was done by BigDye Terminator version 3.1 kits on an ABI 3130 sequencer (Applied Biosystems). Sequences were aligned using BioEdit version 5.0.6 [33]. We tested for neutrality of mutations following Fu & Li's method with D* and F* test statistics using DNASP 4.0 [34,35]. A phylogeny of unique haplotypes was constructed from the calculated Kimura two-parameter distances using the neighbour-joining approach within MEGA ([36]; 1000 bootstrap replicates).

## Competing interests

The author(s) declare that they have no competing interests.

## Authors' contributions

HD collected the majority of the data, and carried out most of the calculations and drafted the text. Y-PM assisted the field work, advised regarding ANOVA's and final edited the text. KD supplied historical information, assisted the field work and advised on all used methods and final edited the text. All three authors read and approved the final text.

## Supplementary Material

Additional file 1 Number of males and females for which body size and wing size is measured and number of individuals used for *IDH1 *allozyme electrophoresis in the Guérande populations. Click here for file

Additional file 2 Number of males and females for which body size and wing size is measured and number of individuals used for *IDH1 *allozyme electrophoresis in the different populations on a European scale. Click here for file

Additional file 3 Allele frequencies from four allozymes (*AO*, *IDH2*, *PGI*, *PGM*) studied in the Guérande populations. *N*: number of studied individuals. Click here for file

Additional file 4 Allele frequencies from four allozymes (*AO*, *IDH2*, *PGI*, *PGM*) studied in the European populations. *N*: number of studied individuals. Click here for file
