# Supplementary material for: Inter- and intraspecific genetic and morphological variation in a sibling pair of carabid species
Source: Saline Syst. 2007 Apr 24;3:4. doi: 10.1186/1746-1448-3-4 (PMC1866230; doi:10.1186/1746-1448-3-4)
Supplement: Additional file 1 — Number of males and females for which body size and wing size is measured and number of individuals used for IDH1 allozyme electrophoresis in the Guérande populations. [file 1746-1448-3-4-S1.doc]

**Appendix 1.** Number of males and females for which body size and wing size is measured and number of individuals used for *IDH1* allozyme electrophoresis in the Guérande populations.

| species | pop |  | Body size / relative wing size | |  | *IDH1* |
| --- | --- | --- | --- | --- | --- | --- |
|  |  |  | male | female |  |  |
| *P. chalceus* | CANAL1 |  | 38 | 28 |  | 67 |
|  | CANAL2 |  | 37 | 28 |  | 67 |
|  | CANAL3 |  | 28 | 22 |  | 51 |
|  | POND1 |  | 58 | 46 |  | 102 |
|  | POND2 |  | 25 | 40 |  | 62 |
|  | POND3 |  | 38 | 17 |  | 57 |
|  |  |  |  |  |  |  |
| *P. littoralis* | GUE1 |  | 13 | 13 |  | 40 |
|  | GUE2 |  | 24 | 14 |  | 39 |
|  | GUE3 |  | 16 | 18 |  | 39 |
