# Supplementary material for: Inter- and intraspecific genetic and morphological variation in a sibling pair of carabid species
Source: Saline Syst. 2007 Apr 24;3:4. doi: 10.1186/1746-1448-3-4 (PMC1866230; doi:10.1186/1746-1448-3-4)
Supplement: Additional file 2 — Number of males and females for which body size and wing size is measured and number of individuals used for IDH1 allozyme electrophoresis in the different populations on a European scale. [file 1746-1448-3-4-S2.doc]

**Appendix 3.** Number of males and females for which body size and wing size is measured and number of individuals used for *IDH1* allozyme electrophoresis in the different populations on a European scale.

| species | pop |  | Body size / relative wing size | |  | *IDH1* |
| --- | --- | --- | --- | --- | --- | --- |
|  |  |  | male | female |  |  |
| *P. chalceus* | FRI |  | 39 | 46 |  | 61 |
|  | BRA |  | 47 | 35 |  | 71 |
|  | WAT |  | 18 | 22 |  | 37 |
|  | MOK |  | 19 | 19 |  | 36 |
|  | ZWC |  | 114 | 127 |  | 261 |
|  | HEI |  | 22 | 26 |  | 47 |
|  | LIS |  | 47 | 30 |  | 76 |
|  | OOS |  | 88 | 81 |  | 161 |
|  | NIE |  | 127 | 171 |  | 265 |
|  | MOE |  | 38 | 40 |  | 73 |
|  | SEA |  | 38 | 28 |  | 60 |
|  | CAN |  | 29 | 19 |  | 47 |
|  | AUT |  | 39 | 40 |  | 77 |
|  | SOM |  | 109 | 92 |  | 185 |
|  | MSM |  | 113 | 111 |  | 221 |
|  | VEY |  | 15 | 18 |  | 33 |
|  | GAC |  | 47 | 29 |  | 35 |
|  | GIR |  | 27 | 15 |  | 41 |
|  | TOU |  | 16 | 12 |  | 30 |
|  | CAM |  | 18 | 9 |  | 29 |
|  | ROU |  | 17 | 16 |  | 27 |
|  | IBI |  | 19 | 10 |  | 30 |
|  | ALB |  | 43 | 24 |  | 30 |
|  | MUR |  | 31 | 20 |  | 30 |
|  | ALM |  | 35 | 12 |  | 30 |
|  |  |  |  |  |  |  |
| *P. littoralis* | ZWC |  | 19 | 15 |  | 48 |
|  | AUT |  | 26 | 25 |  | 46 |
|  | MSM |  | 15 | 10 |  | 40 |
|  | TOU |  | 8 | 7 |  | 72 |
|  | CAM |  | 7 | 19 |  | 40 |
|  | ROU |  | 19 | 19 |  | 30 |
