# Supplementary material for: Inter- and intraspecific genetic and morphological variation in a sibling pair of carabid species
Source: Saline Syst. 2007 Apr 24;3:4. doi: 10.1186/1746-1448-3-4 (PMC1866230; doi:10.1186/1746-1448-3-4)
Supplement: Additional file 3 — Allele frequencies from four allozymes (AO, IDH2, PGI, PGM) studied in the Guérande populations. N: number of studied individuals. [file 1746-1448-3-4-S3.doc]

**Appendix 2.** Allele frequencies from four allozymes (*AO*, *IDH2*, *PGI*, *PGM*) studied in the Guérande populations. *N*: number of studied individuals.

| pop | *N* |  | *AO* | | | | |  | *IDH2* | | |  | *PGI* | | | | | | |  | *PGM* | | | | | |
| --- | --- | --- | --- | --- | --- | --- | --- | --- | --- | --- | --- | --- | --- | --- | --- | --- | --- | --- | --- | --- | --- | --- | --- | --- | --- | --- |
|  |  |  | 1 | 2 | 3 | 4 | 5 |  | 1 | 2 | 3 |  | 1 | 2 | 3 | 4 | 5 | 6 | 7 |  | 1 | 2 | 3 | 4 | 5 | 6 |
| CANAL1 | 75 |  | - | 0.113 | 0.593 | 0.293 | - |  | 0.007 | 0.993 | - |  | - | 0.007 | - | 0.993 | - | - | - |  | - | - | 1 | - | - | - |
| CANAL2 | 70 |  | - | 0.029 | 0.700 | 0.257 | 0.014 |  | 0.007 | 0.993 | - |  | - | 0.036 | - | 0.964 | - | - | - |  | - | - | 1 | - | - | - |
| CANAL3 | 52 |  | 0.010 | 0.183 | 0.519 | 0.279 | 0.010 |  | 0.058 | 0.933 | 0.010 |  | - | 0.010 | 0.010 | 0.981 | - | - | - |  | - | 0.010 | 0.990 | - | - | - |
| POND1 | 107 |  | 0.014 | 0.178 | 0.481 | 0.313 | 0.014 |  | 0.004 | 0.996 | - |  | - | 0.023 | 0.004 | 0.973 | - | - | - |  | - | 0.005 | 0.995 | - | - | - |
| POND2 | 65 |  | 0.031 | 0.077 | 0.623 | 0.262 | 0.008 |  | - | 1 | - |  | - | 0.023 | 0.031 | 0.946 | - | - | - |  | - | - | 0.992 | 0.008 | - | - |
| POND3 | 58 |  | - | 0.250 | 0.560 | 0.190 | - |  | 0.009 | 0.991 | - |  | - | 0.009 | 0.025 | 0.966 | - | - | - |  | - | - | 1 | - | - | - |
|  |  |  |  |  |  |  |  |  |  |  |  |  |  |  |  |  |  |  |  |  |  |  |  |  |  |  |
| GUE1 | 40 |  | - | - | 0.050 | 0.925 | 0.025 |  | - | 1 | - |  | - | 0.338 | - | 0.650 | 0.013 | - | - |  | - | - | - | 0.013 | 0.987 | - |
| GUE2 | 38 |  | - | 0.013 | 0.079 | 0.908 | - |  | 0.013 | 0.987 | - |  | 0.013 | 0.564 | - | 0.423 | - | - | - |  | - | - | 0.026 | 0.051 | 0.923 | - |
| GUE3 | 39 |  | - | 0.013 | 0.141 | 0.846 | - |  | - | 1 | - |  | 0.026 | 0.308 | - | 0.667 | - | - | - |  | - | - | - | - | 1 | - |
