# Supplementary material for: Inter- and intraspecific genetic and morphological variation in a sibling pair of carabid species
Source: Saline Syst. 2007 Apr 24;3:4. doi: 10.1186/1746-1448-3-4 (PMC1866230; doi:10.1186/1746-1448-3-4)
Supplement: Additional file 4 — Allele frequencies from four allozymes (AO, IDH2, PGI, PGM) studied in the European populations. N: number of studied individuals. [file 1746-1448-3-4-S4.doc]

**Appendix 4.** Allele frequencies from four allozymes (*AO*, *IDH2*, *PGI*, *PGM*) studied in the European populations. *N*: number of studied individuals.

| pop | *N* |  | *AO* | | | | |  | *IDH2* | | |  | *PGI* | | | | | | |  | *PGM* | | | | | |
| --- | --- | --- | --- | --- | --- | --- | --- | --- | --- | --- | --- | --- | --- | --- | --- | --- | --- | --- | --- | --- | --- | --- | --- | --- | --- | --- |
|  |  |  | 1 | 2 | 3 | 4 | 5 |  | 1 | 2 | 3 |  | 1 | 2 | 3 | 4 | 5 | 6 | 7 |  | 1 | 2 | 3 | 4 | 5 | 6 |
| FRI | 57 |  | 0.167 | 0.333 | 0.263 | 0.211 | 0.026 |  | - | 0.992 | 0.008 |  | - | 0.009 | 0.009 | 0.982 | - | - | - |  | - | - | 1 | - | - | - |
| BRA | 64 |  | 0.047 | 0.438 | 0.133 | 0.328 | 0.055 |  | - | 1 | - |  | - | - | - | 1 | - | - | - |  | - | - | 1 | - | - | - |
| WAT | 36 |  | 0.319 | 0.319 | 0.278 | 0.056 | 0.028 |  | - | 1 | - |  | - | - | - | 1 | - | - | - |  | - | - | 1 | - | - | - |
| MOK | 39 |  | 0.090 | 0.295 | 0.269 | 0.269 | 0.077 |  | - | 1 | - |  | - | - | 0.100 | 0.900 | - | - | - |  | - | - | 1 | - | - | - |
| ZWC | 110 |  | 0.136 | 0.346 | 0.277 | 0.196 | 0.046 |  | 0.015 | 0.985 | - |  | - | 0.012 | - | 0.988 | - | - | - |  | - | 0.004 | 0.996 | - | - | - |
| HEI | 36 |  | 0.139 | 0.347 | 0.236 | 0.111 | 0.167 |  | - | 1 | - |  | - | - | - | 1 | - | - | - |  | - | - | 1 | - | - | - |
| LIS | 32 |  | 0.250 | 0.094 | 0.359 | 0.156 | 0.141 |  | - | 1 | - |  | - | 0.016 | - | 0.984 | - | - | - |  | - | - | 1 | - | - | - |
| OOS | 114 |  | 0.114 | 0.180 | 0.461 | 0.123 | 0.123 |  | - | 1 | - |  | - | - | - | 1 | - | - | - |  | - | - | 1 | - | - | - |
| NIE | 99 |  | 0.086 | 0.288 | 0.369 | 0.222 | 0.035 |  | 0.004 | 0.996 | - |  | - | - | - | 1 | - | - | - |  | - | - | 1 | - | - | - |
| MOE | 71 |  | 0.042 | 0.282 | 0.338 | 0.197 | 0.141 |  | - | 1 | - |  | - | - | - | 1 | - | - | - |  | - | - | 1 | - | - | - |
| SEA | 79 |  | 0.108 | 0.266 | 0.266 | 0.354 | 0.006 |  | - | 0.994 | 0.006 |  | - | 0.034 | - | 0.966 | - | - | - |  | - | - | 1 | - | - | - |
| CAN | 44 |  | 0.023 | 0.296 | 0.398 | 0.227 | 0.057 |  | - | 0.979 | 0.021 |  | - | - | - | 1 | - | - | - |  | - | 0.028 | 0.972 | - | - | - |
| AUT | 72 |  | 0.063 | 0.313 | 0.313 | 0.278 | 0.035 |  | 0.006 | 0.994 | - |  | - | 0.019 | 0.009 | 0.972 | - | - | - |  | - | - | 1 | - | - | - |
| SOM | 91 |  | 0.121 | 0.302 | 0.346 | 0.187 | 0.044 |  | - | 0.991 | 0.009 |  | - | 0.019 | 0.014 | 0.966 | - | - | - |  | - | 0.005 | 0.995 | - | - | - |
| MSM | 167 |  | 0.129 | 0.476 | 0.270 | 0.111 | 0.015 |  | - | 0.995 | 0.005 |  | - | 0.008 | 0.003 | 0.983 | 0.003 | 0.003 | - |  | 0.005 | - | 0.995 | - | - | - |
| VEY | 33 |  | 0.046 | 0.485 | 0.303 | 0.121 | 0.046 |  | - | 1 | - |  | - | 0.03 | 0.03 | 0.939 | - | - | - |  | - | 0.015 | 0.985 | - | - | - |
| GAC | 34 |  | - | 0.103 | 0.471 | 0.427 | - |  | - | 1 | - |  | - | 0.014 | 0.014 | 0.971 | - | - | - |  | - | - | 1 | - | - | - |
| GIR | 39 |  | 0.051 | 0.205 | 0.282 | 0.462 | - |  | - | 1 | - |  | - | 0.012 | - | 0.988 | - | - | - |  | - | - | 1 | - | - | - |
| TOU | 29 |  | 0.621 | 0.121 | 0.172 | 0.052 | 0.035 |  | - | 1 | - |  | - | 0.033 | 0.067 | 0.833 | 0.05 | 0.017 | - |  | - | - | 0.983 | 0.017 | - | - |
| CAM | 28 |  | 0.661 | 0.089 | 0.179 | 0.071 | - |  | - | 1 | - |  | 0.017 | 0.083 | 0.05 | 0.783 | 0.033 | 0.033 | - |  | - | - | 0.967 | 0.033 | - | - |
| ROU | 31 |  | 0.5 | 0.210 | 0.177 | 0.065 | 0.048 |  | - | 1 | - |  | 0.016 | 0.113 | 0.032 | 0.758 | 0.048 | 0.032 | - |  | 0.048 | - | 0.919 | 0.032 | - | - |
| IBI | 25 |  | 0.74 | 0.16 | 0.08 | 0.02 | - |  | - | 0.983 | 0.017 |  | - | - | 0.117 | 0.8 | - | - | 0.083 |  | - | 0.033 | 0.967 | - | - | - |
| ALB | 28 |  | 0.161 | 0.357 | 0.232 | 0.071 | 0.179 |  | 0.033 | 0.967 | - |  | - | - | 0.017 | 0.95 | - | 0.033 | - |  | - | 0.017 | 0.983 | - | - | - |
| MUR | 30 |  | 0.517 | 0.25 | 0.2 | 0.017 | 0.017 |  | - | 1 | - |  | - | 0.083 | 0.033 | 0.85 | 0.017 | 0.017 | - |  | - | 0.033 | 0.95 | 0.017 | - | - |
| ALM | 27 |  | 0.796 | 0.148 | 0.056 | - | - |  | - | 0.983 | 0.017 |  | - | - | 0.017 | 0.933 | - | 0.05 | - |  | - | 0.033 | 0.95 | 0.017 | - | - |
|  |  |  |  |  |  |  |  |  |  |  |  |  |  |  |  |  |  |  |  |  |  |  |  |  |  |  |
| ZWC | 48 |  | - | - | - | 0.948 | 0.052 |  | - | 1 | - |  | - | 0.188 | - | 0.813 | - | - | - |  | - | - | - | - | 1 | - |
| AUT | 63 |  | - | - | 0.056 | 0.921 | 0.024 |  | - | 1 | - |  | - | 0.315 | - | 0.685 | - | - | - |  | - | - | - | 0.008 | 0.992 | - |
| MSM | 40 |  | - | - | 0.075 | 0.888 | 0.038 |  | 0.013 | 0.988 | - |  | 0.013 | 0.449 | - | 0.539 | - | - | - |  | - | - | 0.013 | 0.025 | 0.95 | 0.013 |
| TOU | 44 |  | - | 0.091 | 0.148 | 0.716 | 0.046 |  | 0.058 | 0.933 | 0.010 |  | 0.089 | 0.644 | - | 0.267 | - | - | - |  | - | - | - | 0.044 | 0.956 | - |
| CAM | 81 |  | - | 0.056 | 0.265 | 0.667 | 0.012 |  | - | 1 | - |  | 0.126 | 0.724 | - | 0.149 | - | - | - |  | - | - | - | 0.081 | 0.908 | 0.012 |
| ROU | 49 |  | - | 0.082 | 0.143 | 0.776 | - |  | - | 1 | - |  | 0.153 | 0.694 | - | 0.153 | - | - | - |  | - | - | - | 0.082 | 0.908 | 0.010 |
